# Treatment of indolent systemic mastocytosis with sarilumab is not supported in a randomized trial

**DOI:** 10.1016/j.jacig.2025.100498

**Published:** 2025-05-21

**Authors:** Hirsh D. Komarow, Jing Wang, Robin Eisch, Linda Scott, Erica H. Brittain, Dean D. Metcalfe

**Affiliations:** a Laboratory of Allergic Diseases, National Institute of Allergy and Infectious Diseases (NIAID), National Institutes of Health (NIH), Bethesda, Md; b Clinical Monitoring Research Program Directorate, Frederick National Laboratory for Cancer Research, Frederick, Md; c Biostatistics Research Branch, Division of Clinical Research, NIAID, NIH, Bethesda, Md

**Keywords:** Mastocytosis, mast cells, tryptase, anti–IL-6, sarilumab

## Abstract

**Background:**

Indolent systemic mastocytosis is a clonal mast cell disease that results in an increase in mast cells in the skin, bone marrow, and other organ systems. IL-6 has been shown to promote mast cell maturation, proliferation, and reactivity *in vitro*. Serum levels of IL-6 correlate with severity of disease and risk of progression of systemic disease.

**Objective:**

We conducted a double-blind placebo-controlled study to assess safety and efficacy for the use of sarilumab in improving the quality of life for those with indolent systemic mastocytosis. ClinicalTrial.gov registration NCT03770273.

**Methods:**

A double-blind trial randomized 16 participants. The primary analysis compared the arms on the Mastocytosis Quality of Life Questionnaire (MC-QoL) index at 16 weeks, adjusting for the baseline MC-QoL. Mean baseline MC-QoL was 47.8.

**Results:**

The difference between the arms in the primary analysis was not statistically significant, with the results favoring the placebo arm; mean absolute MC-QoL improvement in the placebo arm was 14.7 relative to 8.9 in the sarilumab arm. The estimated treatment effect from regression analysis was a 6.0-unit advantage of MC-QoL in the placebo arm (*P* = .40; 95% confidence interval, −8.9, 20.8), limiting a possible sarilumab advantage to at most 9 units of MC-QoL. Similar conclusions were observed for other quality-of-life indices.

**Conclusions:**

Results in this small trial did not support using sarilumab to treat this population and highlights the importance of double-blind randomized studies.

Mastocytosis is clonal disorder of mast cell (MC) proliferation that results in an increase in MCs in the skin, bone marrow (BM), and other organ systems.^1^ Clinical features of mastocytosis include flushing, pruritus, abdominal pain, diarrhea, hypotension, syncope, and musculoskeletal pain. These features are attributed to both the local and systemic release of MC mediators. More than 90% of adult patients with indolent systemic mastocytosis (ISM) have an activating mutation in *KIT,* the receptor for stem cell factor, which results in ligand-independent activation of *KIT.* Life-threatening forms of mastocytosis, including aggressive mastocytosis, systemic mastocytosis associated with a hematologic neoplasm, and MC leukemia, are treated with cytoreductive therapy.

ISM, in contrast, is a less severe systemic variant, and patients with ISM are generally managed over the long term with strategies that offer symptomatic relief. However, symptomatic therapy may be unsuccessful in controlling symptoms, and in many cases, disease continues to progress with both an increase in MC burden and symptoms.

IL-6 is a pleiotropic cytokine involved in inflammation, infection, and cancer. IL-6 is produced by a number of cells including T cells, MCs, and macrophages in response to infection and acute inflammation.^2^ It facilitates interaction between stromal and immune cells, and it has been associated with the pathogenesis of several human MC–related diseases.^3^ IL-6 levels in peripheral blood have been correlated with severity of disease not only in systemic mastocytosis but also in acute and chronic urticaria as well as asthma.^4^, ^5^, ^6^, ^7^, ^8^ Increased IL-6 levels in mastocytosis are correlated with BM pathology, hepatosplenomegaly, osteoporosis, levels of tryptase,^4^ and risk of systemic disease progression.^7^ *In vivo,* IL-6 has also been shown to promote MC maturation, proliferation, reactivity, and downregulation of the soluble IL-6 receptor (IL-6R), thus promoting inflammation,^9^ and IL-6 inhibition reduced oxidative stress in mutant MCs.^10^ Treatment of ISM with an IL-6 antagonist is thus a logical approach to explore toward decreasing MC mediator release and the associated symptoms.

On the basis of the experience of one patient with ISM who tolerated an IL-6 receptor antagonist and whose disease showed a dramatic clinical response based on quality-of-life measurements using the Mastocytosis Quality of Life Questionnaire (MC-QoL), we hypothesized a 60% improvement in MC-QoL in individuals with ISM treated with sarilumab, versus 10% improvement in controls with ISM—a degree of improvement noted to be clinically meaningful in other studies using IL-6 inhibition.^11^ We thus embarked on a double-blind placebo-controlled study to assess the efficacy and safety of sarilumab in the treatment of ISM.

## Methods

### Trial design and oversight

In this randomized, placebo-controlled, double-blind study, we compared sarilumab to placebo in patients with ISM; participants were randomized 1:1 to the two arms. All patients provided informed consent and were enrolled onto the National Institutes of Health/National Institute of Allergy and Infectious Diseases protocol 19-I-0027, “A Phase 2 Randomized Double-Blinded Placebo-Controlled Study to Evaluate the Safety and Efficacy of Subcutaneous Sarilumab in Improving the Quality of Life in Participants with Indolent Systemic Mastocytosis” (NCT03770273, ClinicalTrials.gov). Those randomized to receive the study drug were to be administered 200 mg via subcutaneous injection once every 2 weeks, 8 doses, for a total of 16 weeks. The other participants were to receive a placebo, also administered via subcutaneous injection, every 2 weeks for 16 weeks. All cases were diagnosed as ISM on the basis of World Health Organization criteria, and patients had a baseline MC-QoL of at least 25%. In addition, patients were required to have an absolute neutrophil count of ≥2000/μL, a platelet count of >150,000/μL, and liver transaminases of <1.5 times the upper limit of normal. Patient were excluded from participation if they had an abnormality that would be scored as a grade 4 toxicity on the Common Terminology Criteria for Adverse Events (aka CTCAE) version 5.0, significant abnormalities in the laboratory results, a diagnosis of lymphoma, solid tumors being actively treated, or receipt of any other anti–IL-6 agent or cytoreductive agent within 1 year before the date informed consent was obtained.

Participants were scheduled for a follow-up visit 2 weeks after the final dose (treatment peak) and then again 12 weeks later (washout). Evaluations at study visits included quality of life (QoL) and symptom assessments and measurement of serum tryptase levels and other markers of MC burden. BM examination was performed at the beginning and end of the study. After the week 28 visit, all participants had the option to continue receiving therapy with sarilumab for 52 more weeks, at 200 mg administered via subcutaneous injection. Participants were monitored at each clinical visit for safety concerns.

As developed by Sanofi/Genzyme, sarilumab is a fully human anti–IL-6Rα monoclonal antibody that binds membrane-bound and soluble human IL-6R and inhibits IL-6 signaling.^12^ In 2017, the US Food and Drug Administration approved sarilumab for the treatment of rheumatoid arthritis. The drug’s mechanism of action, in addition to its application in related inflammatory disorders^13^ (eg, ankylosing spondylitis^14^ and uveitis^15^), positions it as a plausible candidate for treatment of patients with ISM.

### Randomization and blinding

Patients were randomized 1:1 to receive 200 mg by subcutaneous injection. If a patient was to exhibit specific adverse effects (ie, cytopenias, liver transaminase elevation) as detailed in the package insert, dosing could be reduced to 150 mg. Patients and clinical and laboratory staff remained unaware of the individual treatment designations. An independent data monitoring committee reviewed unblinded safety data throughout the study. In addition, the Data and Safety Monitoring Board (DSMB) reviewed efficacy data by arm in a one-time futility analysis. The study team statistician was unblinded at this time to prepare the futility analysis report; this statistician was completely independent of the trial’s clinical operations.

### Outcomes

The primary efficacy end point was QoL at 16 weeks after randomization, as measured by the Mastocytosis Quality of Life Questionnaire (MC-QoL).^16^ The primary safety end point was frequency and severity of adverse events (AEs) during the placebo-controlled phase of the trial.

Secondary end points included percentage improvement in MC-QoL from baseline to 16 weeks, and likewise for other QoL assessments: the Memorial Symptom Assessment Scale,^17^ the Mastocytosis Quality-of-Life Questionnaire (MQLQ), and the Mastocytosis Symptoms Assessment Form.^18^ Other secondary end points included reduction in tryptase, allelic frequency of *KIT* D816V, and infiltrating MCs in BM.

### Statistical methods

Several study populations are defined. The modified intent-to-treat (mITT) population includes all participants who received at least one dose of sarilumab or placebo, which in this case is all randomized participants. The per-protocol population only includes those participants who had available data on 16-week MC-QoL and who received >80% of study drug.

The primary analysis compared the groups with respect to the primary end point, the 16-week MC-QoL score in the mITT population using linear regression with an indicator for treatment group and the baseline MC-QoL value as a covariate (the test of the treatment effect has a 2-sided .05 alpha level). While the goal was that all participants will have a 16-week MC-QoL regardless of whether they continued to receive the drug throughout, the last observed value of MC-QoL was carried forward to serve as the primary end point in the primary analysis for those participants without 16-week values. (This includes carrying the baseline value forward if there are no postbaseline measurements.) The planned primary analysis, using the last observation carried forward, implicitly assumed that study participants who stop receiving QoL assessments will be in a stable state, such that the last observed measurement is a reasonable imputation for the 16-week visit. We repeated the regression analysis, but with a log transformation to both QoL values, and percentage change; both analyses included the baseline covariate (which was log-transformed in the case of the log-transformed end point). Analyses were repeated for other QoL indices and for tryptase.

AEs were summarized by study arm, type, severity, and possible relatedness to study drug.

Analyses were conducted by R v4.3.1 software (www.r-project.org). *P* < .05 was considered statistically significant.

We hypothesized a 60% decrease in MC-QoL from baseline to week 16 in the treated population and 10% in placebo as a clinically meaningful difference. We note that this hypothesized treatment effect corresponds to a 0.44 ratio of treatment arm to placebo arm at week 16, which, in turn, corresponds to the drug arm’s having a 56% lower MC-QoL at 16 weeks than placebo. The sample size was originally set to be 15 per arm, as this was the largest feasible. Estimated power for this sample size was based on a simulation of the primary analysis generating baseline and 16-week MC-QoL values modeled on data presented in Siebenhaar et al.^16^ The simulation indicated >90% power with this sample size, allowing some margin for incorrect assumptions.

After experiencing slow recruitment over several years and during the coronavirus disease 2019 pandemic, the DSMB recommended considering a smaller sample size and adding a futility analysis, which had not been included in the original protocol. Power was still estimated to be above 90% under the original assumptions with 10 subjects per arm, and was sufficiently high with alternate modeling of change, so the sample size goal was reduced to a total of 20. A futility analysis was then prespecified by the blinded study team; it was to occur after 12 participants had completed follow-up (ie, reached the week 16 assessment without withdrawing from treatment early). The futility analysis sought to determine if conditional power was >20% (ie, a >20% chance of obtaining a statistically significant result on the primary analysis if the study continued until the planned end, assuming the remainder of the data followed the originally assumed hypothesis). If this was estimated to be a <20% chance, then the DSMB could recommend stopping the study for futility.

## Results

### Demographics

Table I lists baseline characteristics by arm. They were generally comparable by arm, although the placebo participants had a median age of 57 years relative to 47 years in the drug arm. All patients were diagnosed with ISM according to the World Health Organization criteria for systemic mastocytosis.^19^ Markers of MC disease including levels of serum tryptase, percentage *KIT* D816V, percentage MCs in BM, hepatosplenomegaly, and diagnosis of osteopenia/porosis did not differ significantly between the drug and placebo arms. In addition, baseline QoL indices were very similar.

**Table I:** Baseline demographics and markers of disease

Characteristic	Overall	Drug	Placebo	*P* value∗
No. of subjects	15	8	7	
Demographic				
Age (years) at study	47 (44.0-61.5)	46.5 (43.0-47.8)	57 (47.0-67.0)	.13
Sex				
Female	7/15 (46.7)	5/8 (62.5)	2/7 (28.6)	.32
Male	8/15 (53.3)	3/8 (37.5)	5/7 (71.4)	
BMI (kg/m^2^)	28.6 (26.2-32.2)	31.9 (28.4-34.8)	26.2 (25.7-27.9)	.09
Diagnosis (all ISM)				
ISM with UP	15/15 (100%)	8/8 (100%)	7/7 (100%)	1.00
ISM without UP	0	0	0	
Markers of MC disease				
Tryptase (ng/mL)	66.4 (27.0-91.0)	54.5 (26.3-70.4)	89.1 (32.0-106.0)	.40
D816V % in blood	1.1 (0.3-4.7)	0.4 (0.1-4.6)	1.6 (0.6-7.4)	.46
D816V % in BM	0.8 (0.3-5.7)	0.4 (0.2-5.4)	2.1 (0.7-8.6)	.28
D816V mutation	15/15 (100)	8/8 (100)	7/7 (100)	1.00
BM (% MC)	10 (8.5-25.0)	10 (9.3-17.5)	15 (8.5-27.5)	.64
BM aggregates	13/15 (86.67%)	6/8 (75%)	7/7 (100%)	.47
BM spindle shape	14/15 (93.33%)	8/8 (100%)	6/7 (85.71%)	.47
BM CD25 (no. positive/no. reported)	8/8 (100%)	5/5 (100%)	3/3 (100%)	1.00
CD2 flow	15/15 (100%)	8/8 (100%)	7/7 (100%)	1.00
CD25 flow	15/15 (100%)	8/8 (100%)	7/7 (100%)	1.00
Hepatomegaly	6/15 (40%)	2/8 (25%)	4/7 (57.14%)	.32
Splenomegaly	5/15 (33.33%)	4/8 (50%)	1/7 (14.29%)	.28
Osteoporosis	2/15 (13.33%)	0/8 (0%)	2/7 (28.57%)	.20
Osteopenia	6/15 (40%)	2/8 (25%)	4/7 (57.14%)	.32
Total tryptase genotype (copy number)	4.0 (4.0-4.0)	4.0 (4.0-4.0)	4.0 (4.0-4.0)	>.99
IL-6 (pg/mL)	1.6 (1.2-2.4)	1.8 (1.6-2.6)	1.4 (1.1-1.9)	.336
QoL scores				
MC-QoL	41 (34.5-52.5)	39.5 (35.8-53.8)	45 (34.5-52.5)	>.99
MQLQ	120 (84.0-130.5)	122.5 (85.0-132.8)	120 (95.0-128.0)	.64
MSAF	63 (48.0-85.5)	63 (49.5-81.3)	69 (52.5-85.5)	>.99
MSAS	0.9 (0.7-1.2)	0.8 (0.5-1.1)	0.9 (0.7-1.2)	.87
GDI	0.4 (0.3-0.8)	0.4 (0.3-0.8)	0.4 (0.3-0.8)	>.96

Data are presented as n/N (%) for binary variables; and median (interquartile range) for continuous variables. Sample sizes are 7 for placebo and 8 for sarilumab for all comparisons, except 7 for sarilumab for D816V percentage in blood, and as shown for BM CD25. *GDI,* Global Distress Index; *MSAF,* Mastocytosis Symptom Assessment Form; *MSAS,* Memorial Symptom Assessment Scale; *UP,* urticaria pigmentosa.

∗ Wilcoxon rank sum test; Fisher exact test.

**Fig 1:**
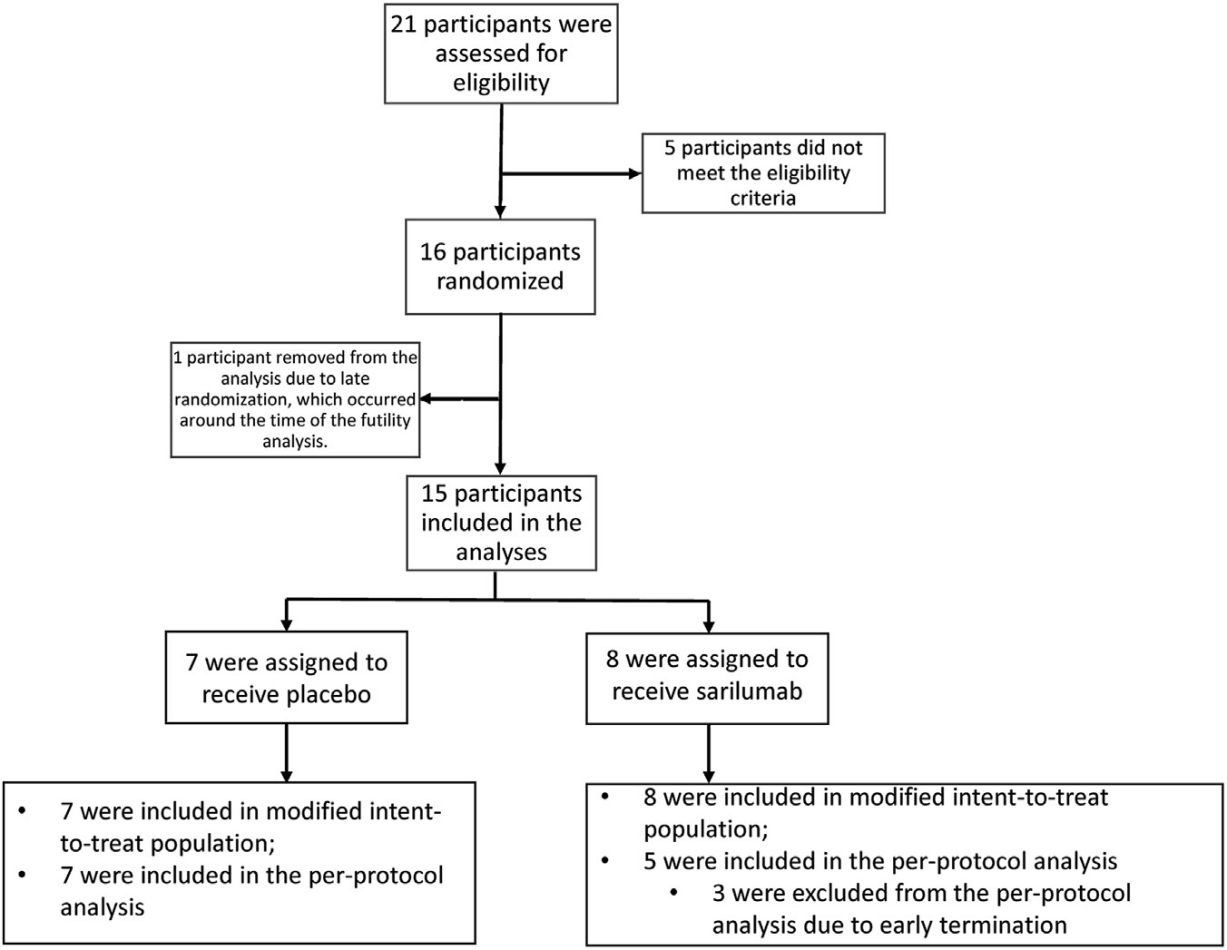
CONSORT (Consolidated Standards of Reporting Trials) diagram.

Twenty-one participants were screened, with 16 randomized. The study’s CONSORT diagram is shown in Fig 1. Five participants were screened but did not meet entry criteria. Eight participants were randomized to each arm.

The study was terminated early after the DSMB recommended the study be discontinued after seeing the results of the futility analysis, which indicated there was <20% probability of finding a statistically significant result on the primary analysis if the study continued to the planned end. The futility analysis included all data from the first 15 participants, 3 of whom withdrew early from drug treatment.

Even though all 16 randomized participants technically met the requirements for the mITT population, the efficacy analyses we present here are based on the 15 participants included in the futility analysis. The final randomized participant (who was assigned to placebo) was not included in the efficacy analyses because the futility analysis was conducted soon after randomization, and no postbaseline values were observed before the participant was notified that the study had been determined to be futile; we therefore thought that this participant did not have the opportunity to provide unbiased data.

Three additional participants withdrew from treatment early from among the 8 assigned to the drug arm. There were no withdrawals among the 8 participants in the placebo arm, except for the final participant randomized after the trial was deemed futile. One of the drug arm withdrawals appeared unrelated to partaking in the trial, as the participant expressed concern about the study appointments interfering with their work schedule. However, the other two withdrawals experienced grade 3 events before withdrawing from the drug. All 16 randomized participants are included in the safety analyses.

Seven participants continued to the open-label portion of the study.

### Efficacy results

Among the 15 participants included in the efficacy analysis, 7 received placebo and 8 received sarilumab. Baseline characteristics are presented by arm in Table I. Overall, the demographic breakdown included 7 female (47%) and 8 male (53%) subjects with an average age of 51.33 years. Median body mass index (BMI) was 28.6 kg/m^2^, and median serum tryptase level was 66.4 ng/mL. Participants in the placebo arm exhibited older median age (57.0 vs 46.5 years), lower median BMI (26.2 vs 31.9 kg/m^2^), and higher median tryptase level (89.1 vs 54.5 ng/mL).

### Primary analysis

The primary analysis compared the treatment group in terms of the primary end point, MC-QoL, with baseline MC-QoL as the covariate in the mITT population. Treatment effects are shown in Table II, and corresponding raw comparative data are listed in Table III. All 15 participants in the efficacy analyses are included in the mITT analyses; the two without week 16 values had the last observation carried forward in the primary analysis, as prespecified in the protocol.

**Table II:** Means and treatment effect adjusted for baseline covariate of study end points by treatment group

End point at 16 weeks	Population	Mean of raw data						Linear regression results†				
		Placebo			Sarilumab			Drug LSM	Placebo LSM	Treatment estimate	Treatment 95% confidence interval	*P* value‡
		Baseline	Week 16	Change∗	Baseline	Week 16	Change∗					
MC-QoL	mITT	47.71	33	14.71	47.88	39	8.88	38.98	33.02	5.95	−8.91, 20.82	.3998
Log MC-QoL	mITT	3.87	3.50	0.37	3.87	3.66	0.21	3.64	3.42	0.22	−0.32, 0.76	.3943
%Change MC-QoL§	mITT	—	—	30.83†	—	—	18.55†	15.18	20.64	−5.45	−45.90, 35.00	.7740
MC-QoL	PP	47.71	33.0	14.71	52.40	40.2	12.20	39.50	33.49	6.01	−13.14, 25.15	.4959
MQLQ	mITT	124.14	99.86	24.29	125.50	111.38	14.12	111.02	100.26	10.76	−22.20, 43.72	.4904
MSAF	mITT	73.43	42.29	31.14	71.62	55.75	15.88	55.83	42.20	13.63	−5.16, 32.42	.1399
MSAS	mITT	0.91	0.51	0.40	0.86	0.76	0.10	0.767	0.503	0.26	−0.07, 0.59	.1071
GDI	mITT	0.54	0.28	0.26	0.52	0.46	0.06	0.458	0.279	0.18	−0.10, 0.46	.1890
Tryptase (ng/mL)	mITT	79.87	84.93	−5.06	53.09	56.56	−3.47	69.20	70.49	−1.30	−12.06, 9.46	.7973

*GDI,* Global Distress Index; *LSM,* least square means; *MSAF,* Mastocytosis Symptom Assessment Form; *MSAS,* Memorial Symptom Assessment Scale; *PP,* per protocol.

∗ Change refers to baseline mean minus week 16 mean.

† LSM are adjusted for baseline covariate, where baseline covariate is baseline QoL score.

‡ *P* value testing treatment effect in linear regression model.

§ Percentage change (%Change) is presented instead of absolute change, where %Change = [(baseline − week 16) × 100]/baseline.

**Table III:** Baseline versus treatment peak results

Characteristic	Placebo (n = 7)			Drug (n = 8)			*P* value†
	Baseline	Week 16	Change∗	Baseline	Week 16	Change∗	
Markers of MC disease							
Tryptase (ng/mL)	89.1 (32, 106)	99.9 (33.9, 117.5)	−3.2 (−9.0 to 0.2)	54.5 (26.3, 70.4)	58.2 (29.1, 76.1)	−0.4 (−4.9 to 0.5)	.64
D816V % in blood	1.6 (0.6, 7.4)	1.2 (0.5, 8.3)	0.2 (0.0 to 0.3)	0.4 (0.1, 4.6)	3.3 (0.2, 5.5)	0.0 (−0.7 to 0.2)	.43
D816V % in BM	2.1 (0.7, 8.6)	1.8 (0.9, 9.7)	0.1 (−0.2 to 0.2)	0.4 (0.2, 5.4)	0.3 (0.1, 0.4)	0.1 (0.0 to 0.2)	.88
BM (% MC)	15 (8.5, 27.5)	15 (6.5, 25)	2.0 (−2.5 to 5.0)	10 (9.2, 17.5)	15 (10, 25)	0	.73
BM aggregates	7/7 (100%)	7/7 (100%)	0	6/8 (75%)	6/8 (75%)	0	1
BM spindle shape	6/7 (85.7%)	6/7 (85.7%)	0	8/8 (100%)	8/8 (100%)	0	1
BM CD25, no. positive/no. reported (%)	3/3 (100%)	3/3 (100%)	0	5/5 (100%)	5/5 (100%)	0	1
CD2 flow positive	7/7 (100%)	7/7 (100%)	0	8/8 (100%)	8/8 (100%)	0	1
CD25 flow positive	7/7 (100%)	7/7 (100%)	0	8/8 (100%)	8/8 (100%)	0	1
Liver size (cm)	17 (15.9, 18.3)	16.8 (15.4, 17.5)	0.3 (−0.5 to 0.5)	17.2 (15.8, 17.7)	16.4 (15.6, 18)	−0.4 (−0.8 to 0.3)	.72
Spleen size (cm)	11.8 (11.4, 12.5)	12 (10.9, 13.4)	−0.3 (−0.5 to 0.5)	13.1 (11.8, 14.1)	13.5 (12.3, 14)	0.5 (−0.5 to 1.6)	.34
Relevant laboratory results							
ALK (U/L)	87 (85, 92.5)	92 (83.5, 96.5)	−4.0 (−4.0 to 4.5)	91 (66.2, 104.5)	65 (64.5, 74.5)	25.0 (−0.5 to 26.5)	.14
CRP (mg/L)	2.4 (1.6, 3.6)	2.2 (1.6, 4.3)	0.2 (−0.3 to 0.3)	6.5 (2.8, 9.1)	2.9 (1.5, 4.7)	0.6 (0.2 to 4.2)	.13

For continuous variables, medians (interquartile ranges) are reported by baseline and week 16. For binary variables, n/N (%) are reported by baseline and week 16. *ALK,* Alkaline phosphatase; *CRP,* C-reactive protein.

∗ Change refers to baseline mean minus week 16 mean.

† For continuous variables, *P* values are extracted from Wilcoxon rank sum tests comparing difference in change from baseline to week 16 by treatment arms. For binary variables, *P* values are from Fisher exact tests testing proportion of switching status from baseline to week 16 between treatment arms. No imputation was done for missing data.

Table II shows these results: the adjusted 16-week mean MC-QoL is 38.98 for drug and 33.02 for placebo, where low values signify less impairment; this corresponds to a 5.95 treatment difference on week 16 MC-QoL. While the result does not show a statistically significant difference between the arms (*P* = .40), the results favor the placebo arm, with on average about a 6-unit better MC-QoL score. Because the study is small, the confidence interval for the treatment effect is wide, and a modest drug benefit cannot be ruled out. That is, the lower limit of the confidence interval for the treatment effect indicates we cannot rule out a 9-unit benefit in the drug arm relative to placebo; however, on the basis of the upper limit, it is possible there a 21-unit deficit relative to placebo.

**Fig 2:**
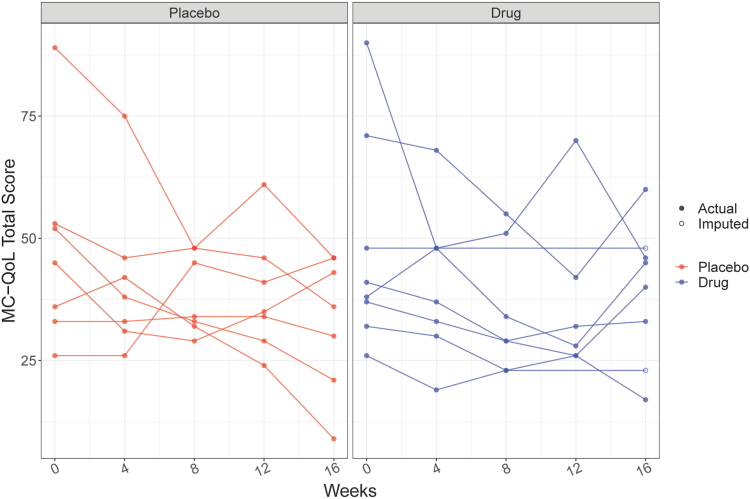
MC-QoL score over time by treatment. Change of MC-QoL score at weeks 0, 4, 8, 12, and 16 by treatment arm (*left,* placebo; *right,* drug). Observed data are shown in *solid circles;* imputed data, *empty circles.*

Fig 2 illustrates the results graphically, showing 3 of 7 placebo participants with noteworthy improvements in MC-QoL, represented by downward slopes. These 3 individuals all experienced >60% improvement in the MC-QoL. In contrast, none of the 8 treated participants experienced a 60% improvement, although one had an approximate 50% change. The radar plot (see Fig E1 in the Online Repository available at www.jaci-global.org) also suggests a greater improvement from baseline observed in the placebo participants in both the skin and symptom subdomains of the MC-QoL.

### Supportive and secondary analyses

Similar results are seen in Table II for analogous MC-QoL analysis on the log-transformed scale, for percentage change in MC-QoL, and of MC-QoL in the per-protocol population. All results are similar to the primary analysis in that none is statistically significant, and all favor the placebo arm, with better mean values for week 16 log MC-QoL, greater mean percentage change, and better mean values for week 16 MC-QoL in the per-protocol population. All associated confidence intervals are wide. The planned mixed-effect model (a sensitivity analysis) was omitted because of the smaller-than-planned sample size.

The analysis of MC-QoL on the log-transformed scale provides an estimate of treatment effect that uses the same scale as the hypothesized effect. As described in the sample size description, the hypothesized treatment effect corresponds to the drug arm’s having a 56% better MC-QoL at 16 weeks than placebo. However, when the observed treatment effect shown for log MC-QoL in Table II is exponentiated, the point estimate of the ratio of drug to placebo is 1.24, which corresponds to the drug’s leading to a 24% worse week 16 MC-QoL relative to placebo. Again, the confidence interval is wide, and the optimistic end of the exponentiated confidence interval corresponds to a 27% better MC-QoL in the treated arm than placebo. To sum up, the log-transformed analysis favors placebo, and although results were not statistically significant, the analysis indicates that the drug arm MC-QoL could be at most 27% better than the placebo arm at week 16.

The analysis of the 4 other QoL indices in the mITT analysis provide similar outcomes (Table II). All results favor the placebo arm, and while there are no statistically significant differences between the two arms, the *P* values are somewhat smaller for the Mastocytosis Symptoms Assessment Form, the Memorial Symptom Assessment Scale, and the Global Distress Index indices than for MC-QoL, suggesting more evidence of little to no efficacy of the drug.

Tryptase values were somewhat different by arm at baseline, with the mean placebo of 79.87 and mean drug value of 53.09, with little change observed in means at week 16 in either arm. This is the only analysis presented in Table II that does not favor placebo, but the estimated treatment effect is very small, with adjusted week 16 means of 69.20 for drug and 70.49 for placebo, and not statistically significant (*P* = .797).

Other markers of MC disease, including percentage *KIT* D816V in peripheral blood and BM, percentage MC in BM, MC morphology, size of liver and spleen, and presence of markers of MC clonality (CD2 and CD25), changed minimally, if at all, from baseline in both the drug and placebo groups. There was some indication of a decline in other markers of inflammation (alkaline phosphatase, C-reactive protein) in the drug arm, but this did not reach significance (Table III). Similarly, other laboratory results including complete blood count and differential, liver enzyme levels, and bilirubin levels were not significantly changed (see Table E1 in the Online Repository available at www.jaci-global.org).

### Analysis of serum IL-6 levels

Consistent with early reports,^4^ baseline IL-6 levels were found to be significantly elevated in patients over a normal volunteer cohort (see Fig E2 in the Online Repository available at www.jaci-global.org). There is no indication that baseline IL-6 values correlate with baseline MC-QoL or change in MC-QoL (see Fig E3 in the Online Repository). While the ability to consider the treatment effect as a function of baseline IL-6 is limited given the small sample size, there is no suggestion that there is an IL-6 subgroup where the disease of treated participants did well relative to placebo recipients.

### Safety

All 16 randomized participants were included in the safety tables (Table IV; and see Tables E2 and E3 in the Online Repository available at www.jaci-global.org). Generally, there was little indication of differences by arm with respect to AEs. In both arms, 7 of 8 participants experienced at least one AE. There was one AE category (yeast infections) where 3 of 8 sarilumab participants had events versus 0 in 8 placebo participants. Furthermore, two participants in the sarilumab arm withdrew after experiencing grade 3 events (Table E3). For one participant, the event was a decrease in neutrophils deemed probably related (the week 16 value was observed after treatment withdrawal), and the other participant had a colon perforation that was deemed possibly related. No placebo participants stopped doses early (except for the single participant who was in early follow-up at the time the study was deemed futile); two experienced a grade 3 event (hypertension), although both events were deemed unrelated to the study drug.

**Table IV:** AEs by MedDRA system organ class, preferred term, and treatment arm

System organ class	Preferred term	All (N = 16)	Placebo (n = 8)	Drug (n = 8)
Blood and lymphatic system disorders	Eosinophilia	1 (1) 6.25%	1 (1) 12.50%	0
Cardiac disorders	Palpitations	1 (1) 6.25%	1 (1) 12.50%	0
Ear and labyrinth disorders	Ear pain	1 (1) 6.25%	0	1 (1) 12.50%
Eye disorders	Eye pain	1 (1) 6.25%	0	1 (1) 12.50%
Gastrointestinal disorders	Colon perforation	1 (1) 6.25%	0	1 (1) 12.50%
	Constipation	1 (1) 6.25%	0	1 (1) 12.50%
	Diarrhea	1 (1) 6.25%	0	1 (1) 12.50%
	Nausea	1 (1) 6.25%	1 (1) 12.50%	0
	Mouth sores	1 (1) 6.25%	0	1 (1) 12.50%
	Vomiting	2 (2) 12.50%	1 (1) 12.50%	1 (1) 12.50%
General disorders and administration site conditions	Chest tightness	1 (1) 6.25%	1 (1) 12.50%	0
	Fatigue	1 (1) 6.25%	1 (1) 12.50%	0
	Flulike symptoms	1 (1) 6.25%	0	1 (1) 12.50%
	Induration (skin and subcutaneous tissue) at injection site	4 (1) 6.25%	0	4 (1) 12.50%
	Injection site bruising	1 (1) 6.25%	0	1 (1) 12.50%
	Injection site erythema	2 (2) 12.50%	0	2 (2) 25.00%
	Injection site reaction	1 (1) 6.25%	1 (1) 12.50%	0
	Injection site redness	1 (1) 6.25%	0	1 (1) 12.50%
	Pain	1 (1) 6.25%	0	1 (1) 12.50%
Immune system disorders	Allergic reaction	1 (1) 6.25%	1 (1) 12.50%	0
Infections and infestations	Bacterial vaginosis	2 (1) 6.25%	0	2 (1) 12.50%
	Bronchial infection	1 (1) 6.25%	0	1 (1) 12.50%
	Urinary tract infection	1 (1) 6.25%	0	1 (1) 12.50%
	Yeast infection	4 (3) 18.75%	0	4 (3) 37.50%
Infections and infestations	Pain at biopsy site	1 (1) 6.25%	0	1 (1) 12.50%
	Postoperative pain	1 (1) 6.25%	1 (1) 12.50%	0
Investigations	Alanine aminotransferase increased	3 (2) 12.50%	0	3 (2) 25.00%
	Aspartate aminotransferase increased	1 (1) 6.25%	0	1 (1) 12.50%
	Blood bilirubin increased	2 (2) 12.50%	0	2 (2) 25.00%
	Chloride increased	1 (1) 6.25%	1 (1) 12.50%	0
	Cholesterol high	2 (2) 12.50%	0	2 (2) 25.00%
	Lymphocyte count decreased	5 (4) 25.00%	4 (3) 37.50%	1 (1) 12.50%
	Neutrophil count decreased	2 (1) 6.25%	0	2 (1) 12.50%
	Platelet count decreased	1 (1) 6.25%	0	1 (1) 12.50%
	Weight loss	1 (1) 6.25%	0	1 (1) 12.50%
	White blood cell decreased	3 (1) 6.25%	0	3 (1) 12.50%
Metabolism and nutrition disorders	Hypercalcemia	1 (1) 6.25%	1 (1) 12.50%	0
	Hyperglycemia	4 (4) 25.00%	2 (2) 25.00%	2 (2) 25.00%
	Hypertriglyceridemia	6 (6) 37.50%	3 (3) 37.50%	3 (3) 37.50%
Musculoskeletal and connective tissue disorders	Knee pain	1 (1) 6.25%	1 (1) 12.50%	0
	Leg pain	1 (1) 6.25%	1 (1) 12.50%	0
	Pain in hip	1 (1) 6.25%	1 (1) 12.50%	0
Nervous system disorders	Dizziness	2 (1) 6.25%	0	2 (1) 12.50%
	Hyperosmia	1 (1) 6.25%	1 (1) 12.50%	0
	Light headedness	6 (1) 6.25%	0	6 (1) 12.50%
	Memory impairment	1 (1) 6.25%	0	1 (1) 12.50%
Psychiatric disorders	Insomnia	5 (3) 18.75%	3 (2) 25.00%	2 (1) 12.50%
	Irritability	1 (1) 6.25%	1 (1) 12.50%	0
Renal and urinary disorders	Urinary retention	1 (1) 6.25%	0	1 (1) 12.50%
Reproductive system and breast disorders	Dysmenorrhea	1 (1) 6.25%	0	1 (1) 12.50%
Respiratory, thoracic, and mediastinal disorders	Cough	1 (1) 6.25%	0	1 (1) 12.50%
	Nasal congestion	1 (1) 6.25%	0	1 (1) 12.50%
	Sore throat	2 (2) 12.50%	0	2 (2) 25.00%
Skin and subcutaneous tissue disorders	Dry skin	1 (1) 6.25%	1 (1) 12.50%	0
	Hives	1 (1) 6.25%	0	1 (1) 12.50%
	Pruritus	3 (1) 6.25%	0	3 (1) 12.50%
	Rash	1 (1) 6.25%	0	1 (1) 12.50%
Social circumstances	Perimenopause	1 (1) 6.25%	1 (1) 12.50%	0
Vascular disorders	Hypertension	11 (10) 62.50%	6 (5) 62.50%	(5) 62.50%

Each field uses format # (X) %, where # is number of AEs, X is number of participants with one or more episodes of given AE, and % is number of participants with one or more episodes of given AE divided by total number of participants who received study agent (N) multiplied by 100. Data of one participant from placebo arm were excluded from statistical analyses.

## Discussion

It is important to carefully interpret the results in the context of this small trial with a negative result. This study provided no support for the use of sarilumab in this mastocytosis population, as all analyses of QoL showed numerically better results in the placebo arm than the drug arm. Nonetheless, because the study is small, confidence intervals for the treatment effect are wide, and there is no statistically significant difference between the arms; thus, it is not possible to rule out a small benefit of sarilumab on MC-QoL. However, the most optimistic limit of the confidence interval for the primary analysis is consistent with any possible benefit of the drug being at most 9 MC-QoL units; further, our data indicate that a benefit of even this small magnitude is unlikely. If the use of this drug would not be warranted for a 9-unit advantage in MC-QoL, then even this small negative trial provides clear evidence against the use of sarilumab in the studied population. Furthermore, 2 of the 8 treated participants experienced grade 3 events that were judged to be possibly (or in one case, probably) related to sarilumab, and they were consequently withdrawn from treatment; another drug arm participant opted to withdraw for apparently unrelated reasons.

As we have already noted, the placebo group fared somewhat better on the QoL indices than the sarilumab group, but this result was not statistically significant and could easily be due to the inherent randomness of this small study. While a detrimental effect of sarilumab on QoL cannot be ruled out (as illustrated by the confidence interval of the treatment difference for the primary end point), the observed difference between arms was not nearly enough to yield a statistical conclusion of detriment, as indicated by the 2-sided *P* value of .40 in the primary analysis. It is also noteworthy that most of the subjects in both arms had better MC-QoL scores at 16 weeks relative to baseline.

None of the 8 treated participants experienced the hypothesized 60% improvement in MC-QoL (although one was close), whereas 3 of 7 placebo participants did experience a benefit of this magnitude. This trial clearly illustrates the advantage of conducting blinded placebo-controlled trials, especially for a subjective end point. Had a one-arm trial been conducted instead, a more optimistic conclusion might have been reached.

This trial also illustrates the power of the placebo effect: several patients receiving placebo reported significant improvement in symptoms consistent with manifestations of ISM in the skin, gastrointestinal tract, and musculoskeletal system. This study highlights the importance of performing blinded and randomized clinical trials that may identify a drug effect that is more significant than placebo. The power of placebo has been identified in numerous clinical trials and is often described as a contextual effect,^20^ which likely played a role in our outcome here, given that the primary outcome was QoL rather than specific markers of MC activation (like tryptase) or changes in BM findings (ie, percentage mass, cells, changes in morphology, frequency of D816V mutation in *KIT* MCs, presence of markers of clonality in CD2 and CD25).

There are several possible mechanisms by which sarilumab is seemingly ineffective in treating ISM. Perhaps sarilumab blockage at the tissue level was insufficient to influence the effect of IL-6 on target cells, and an agent that decreases IL-6 production directly may yield more significant results. Or perhaps the overall effect of targeted blocking of IL-6 in mastocytosis is minimal considering the complexity of the underlying pathologic processes and the influence of many other elevated inflammatory mediators.

With the advent of more precisely targeted cytokine reductive agents in patients with ISM^21^ that may have fewer adverse effects, patients are likely to experience genuine improvement in symptoms that are consistent with a decrease in MC burden.

## Disclosure statement

Supported by the 10.13039/100006492Division of Intramural Research, National Institute of Allergy and Infectious Diseases, 10.13039/100000002National Institutes of Health (NIH). This project was funded in whole or in part with federal funds from the 10.13039/100000054National Cancer Institute, NIH, under contract 75N91019D00024. The content of this publication does not necessarily reflect the views or policies of the Department of Health and Human Services; nor does mention of trade names, commercial products, or organizations imply endorsement by the US government.

Disclosure of potential conflict of interest: The authors declare that they have no relevant conflicts of interest.
