# Supplementary material for: Treatment of indolent systemic mastocytosis with sarilumab is not supported in a randomized trial
Source: J Allergy Clin Immunol Glob. 2025 May 21;4(3):100498. doi: 10.1016/j.jacig.2025.100498 (PMC12172267; doi:10.1016/j.jacig.2025.100498)

**Supplemental Figure 1:** Scores of the 4 Domains Measured in the Mastocytosis Quality of Life Questionnaire at Baseline and Week 16 by Treatment Arm


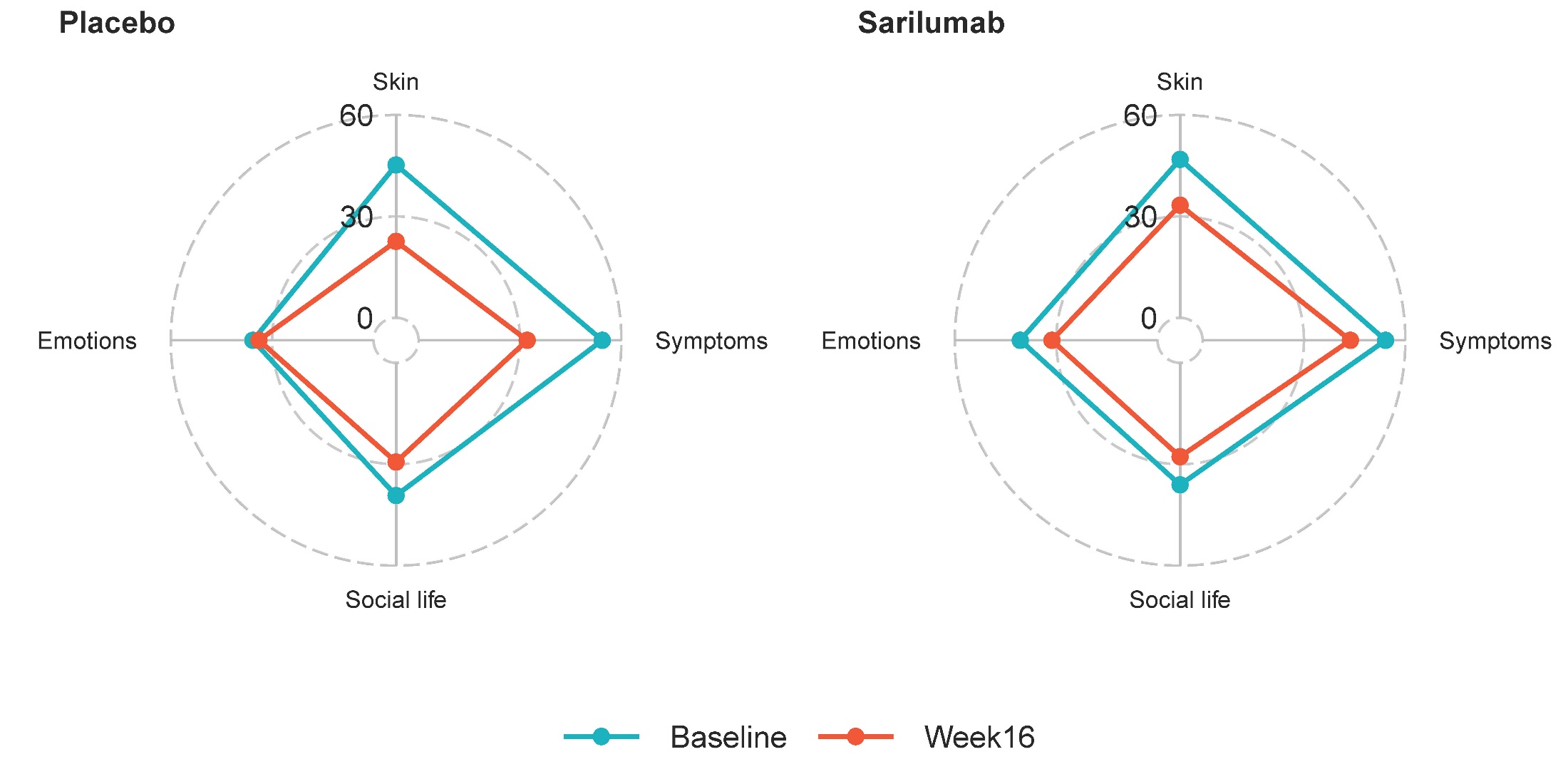


**Supplemental Figure 2: Baseline IL-6 levels**

Baseline serum IL-6 levels in patients were significantly elevated over normal volunteers.

**Supplemental Figure 3:** Scatterplots of (baseline levels of) IL-6 vs. Baseline, Absolute Change and Percent Change of Mc-QoL by Treatment Arm


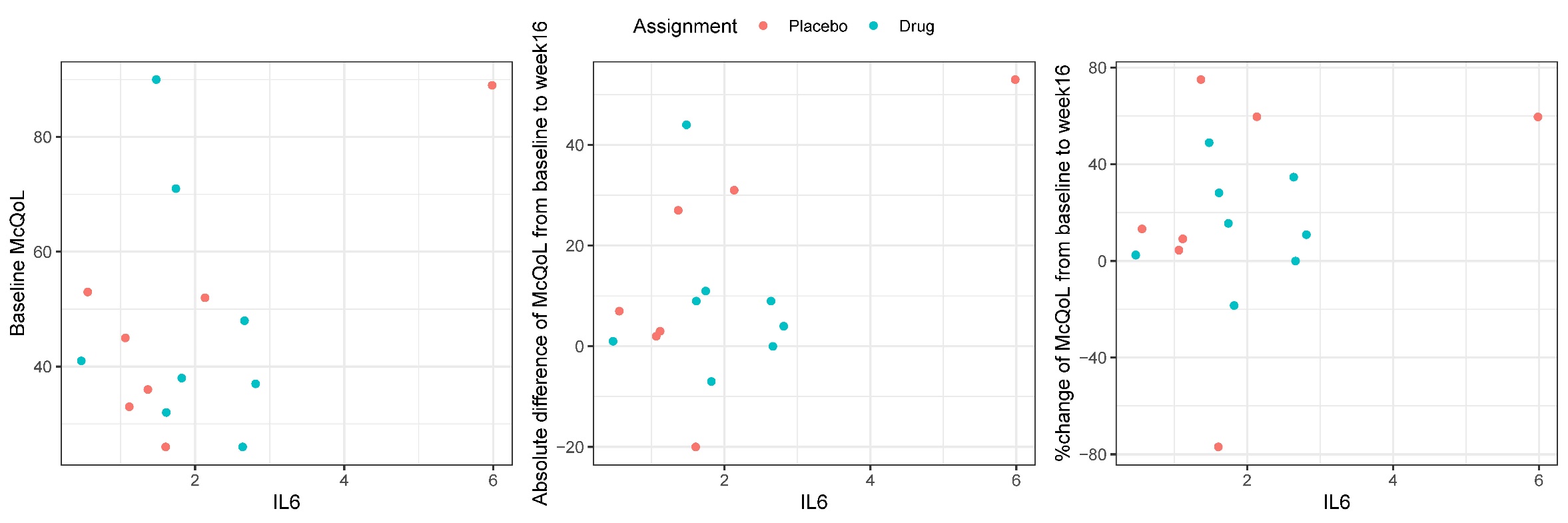

Supplement: Supplementary Figs [file mmc1.docx]
