# Supplementary material for: Treatment of indolent systemic mastocytosis with sarilumab is not supported in a randomized trial
Source: J Allergy Clin Immunol Glob. 2025 May 21;4(3):100498. doi: 10.1016/j.jacig.2025.100498 (PMC12172267; doi:10.1016/j.jacig.2025.100498)
Supplement: Supplementary Tables [file mmc2.docx]

Supplemental Table 1: Lab results Baseline vs. Peak Treatment

|  | **Placebo**  **(N=7)** | | | **Drug**  **(N=8)** | | |  |
| --- | --- | --- | --- | --- | --- | --- | --- |
|  | **Baseline** | **Week 16** | **Baseline – Week 16** | **Baseline** | **Week 16** | **Baseline – Week 16** | **P value**^1^ |
| **White blood cell count (WBC)** (K/mcL) | 6.6 (4.9,7.7) | 6 (5.3,6.7) | 0.3 (0.1-1.2) | 8.6 (6.5,10.1) | 7.5 (6.4,8.8) | 0.2 (-0.8-1.3) | 0.54 |
| **Hemoglobin (HGB)** (g/dL) | 14.1 (13.7,15) | 14.4 (14.1,15.2) | 0.0 (-0.3-0.4) | 13.8 (13.1,15.1) | 13.7 (13.3,14.7) | 0.1 (-0.2-0.4) | 0.65 |
| **Hematocrit (HCT)** (%) | 42.2 (40.6,44.8) | 43.1 (42.2,44.8) | 0.0 (-1.1-2.0) | 41.6 (39.8,43.6) | 40.6 (38.8,43.9) | 0.7 (0.6-1.4) | 0.61 |
| **Platelet count (PLT)** (K/mcL) | 242 (229,270.5) | 227 (217,287.5) | 12.0 (-20.0-16.5) | 290.5 (243.5,316.8) | 227 (219.5,255) | 55.0 (0.0-64.5) | 0.26 |
| **Absolute neutrophil count (ANC)** (K/mcL) | 3.3 (2.7,4.8) | 3.3 (2.7,3.6) | 0.4 (0.2-1.1) | 5.2 (4.3,6) | 4.6 (3.5,5) | 0.5 (-0.6-1.1) | 0.71 |
| **Absolute lymphocyte count (ALC)** (K/mcL) | 1.8 (1.3,1.9) | 1.6 (1.4,2.1) | -0.2 (-0.4-0.2) | 2.4 (2,2.4) | 2.5 (1.9,2.6) | -0.1 (-0.3-0.1) | >0.99 |
| **Absolute eosinophil count (AEC)** (K/mcL) | 0.3 (0.2,0.4) | 0.3 (0.2,0.5) | 0.0 (-0.1-0.1) | 0.2 (0.2,0.3) | 0.3 (0.2,0.4) | 0.0 (-0.1-0.0) | 0.37 |
| **ALT (SGPT)** (U/L) | 23 (13,25.5) | 24 (19.5,28) | -3.0 (-4.0--1.0) | 19 (14.8,25.2) | 20 (15,36.5) | -5.0 (-17.0-2.5) | 0.61 |
| **AST (SGOT)** (U/L) | 14 (13,20.5) | 19 (15,21.5) | -2.0 (-2.0-0.0) | 15 (13.2,16.2) | 14 (13,28.5) | -3.0 (-11.5--0.5) | 0.30 |
| **Total Bilirubin** (mg/dL) | 0.4 (0.3,0.7) | 0.6 (0.4,0.8) | 0.0 (-0.3-0.2) | 0.6 (0.4,0.8) | 0.5 (0.5,0.9) | 0.0 (-0.3-0.2) | >0.99 |
| ^1^P-values are extracted from Wilcoxon rank sum tests comparing the difference in change from baseline to week16 by treatment arms.  Medians (IQRs) are reported by baseline and week16. Baseline – Week16 column shows the median of (baseline – week 16) | | | | | | | |

## Supplemental Table 2: Possibly, Probably, and Definitely Related Adverse Events by MedDRA Lowest Level Term and Treatment Arm

| **MedDRA Lowest Level Term** | **All (N=16)** | **Placebo (N=8)** | **Drug (N=8)** |
| --- | --- | --- | --- |
| Alanine aminotransferase increased | 3 (2) 12.50% | 0 (0) 0% | 3 (2) 25.0% |
| Aspartate aminotransferase increased | 1 (1) 6.25% | 0 (0) 0% | 1 (1) 12.5% |
| Colon perforation | 1 (1) 6.25% | 0 (0) 0% | 1 (1) 12.5% |
| Hypertriglyceridemia | 3 (3) 18.75% | 3 (3) 37.5% | 0 (0) 0% |
| Injection site bruising | 1 (1) 6.25% | 0 (0) 0% | 1 (1) 12.5% |
| Injection site erythema | 2 (2) 12.50% | 0 (0) 0% | 2 (2) 25.0% |
| Injection site induration | 4 (1) 6.25% | 0 (0) 0% | 4 (1) 12.5% |
| Injection site redness | 1 (1) 6.25% | 0 (0) 0% | 1 (1) 12.5% |
| Lymphocyte count decreased | 2 (2) 12.50% | 2 (2) 25.0% | 0 (0) 0% |
| Neutrophil count decreased | 2 (1) 6.25% | 0 (0) 0% | 2 (1) 12.5% |
| Platelet count decreased | 1 (1) 6.25% | 0 (0) 0% | 1 (1) 12.5% |
| White blood cell decreased | 3 (1) 6.25% | 0 (0) 0% | 3 (1) 12.5% |

N: Total number of study participants who received study agent (one participant from placebo arm was excluded from the statistical analyses).

Each field has the format # (X) %, where # is the number of adverse events, X is the number of participants with one or more episodes of the given event, and % is the number of participants with one or more episodes of the given event divided by the total number of participants who received the study agent (N) multiplied by 100.

## Supplemental Table 3: Grade 3 and Above Adverse Events by MedDRA Lowest Level Term and Treatment Arm

| **MedDRA Lowest Level Term** | **All (N=16)** | **Placebo (N=8)** | **Drug (N=8)** |
| --- | --- | --- | --- |
| Colon perforation | 1 (1) 6.25% | 0 (0) 0% | 1 (1) 12.5% |
| Hypertension | 2 (2) 12.50% | 2 (2) 25.0% | 0 (0) 0% |
| Neutrophil count decreased | 2 (1) 6.25% | 0 (0) 0% | 2 (1) 12.5% |

N: Total number of study participants who received study agent (one participant from placebo arm was excluded from the statistical analyses).

Each field has the format # (X) %, where # is the number of adverse events, X is the number of participants with one or more episodes of the given event, and % is the number of participants with one or more episodes of the given event divided by the total number of participants who received the study agent (N) multiplied by 100.
